# Genome-wide identification, characterization, and expression analysis of UDP-glycosyltransferase genes associated with secondary metabolism in alfalfa (*Medicago sativa* L.)

**DOI:** 10.3389/fpls.2022.1001206

**Published:** 2022-09-30

**Authors:** Andong Yu, Xueqian Jiang, Yan Sun, Qiannan Hu, Xiaoxi Zhu, Junmei Kang, Lin Chen, Lin Liu, Linfeng Hao, Qingchuan Yang, Ruicai Long, Mingna Li

**Affiliations:** ^1^Institute of Animal Sciences, Chinese Academy of Agricultural Sciences, Beijing, China; ^2^College of Grassland Science and Technology, China Agricultural University, Beijing, China; ^3^Bayannur Institute of Agricultural and Animal Husbandry Sciences, Inner Mongolia, China

**Keywords:** *Medicago sativa*, UDP glycosyltransferase, secondary metabolism, flavonoids, terpenoids, phylogenetic analysis

## Abstract

Uridine diphosphate glycosyltransferases (UGTs) are enzymes that catalyze glycosylation modifications and play an essential role in regulating plant metabolism. Alfalfa (*Medicago sativa* L.) is the most important legume in the world due to its high yields and protein content; however, the *UGT* genes in alfalfa have not yet been studied. Identifying *UGT* genes with metabolic roles in alfalfa is essential for identifying and modifying genetic traits that are relevant to yield and quality. In this study, 90 of the 239 *UGT* genes identified from the alfalfa “Zhongmu No. 1” genome database were found to be related to secondary metabolism, and a series of gene family characterization analyses were conducted on each. The results demonstrated that all 90 *UGT* genes were unevenly distributed on eight chromosomes with few introns and that tandem duplications were the crucial driving force expanding the UGT family in alfalfa. Notably, the 90 *UGT* genes can be clustered into ten evolutionary groups which contain specific PSPG motifs, and genes in these ten groups have specific tissue expressions. This suggests that the *UGT* genes in each group could have similar glycosylation roles corresponding to analogous secondary metabolites in alfalfa. Additionally, multiple *cis*-acting elements found in *MsUGT* promoter regions, such as phytohormone and flavonoids, indicate that 90 *UGT* members could be induced by these features, which are also related to secondary metabolism. Therefore, our study identified 90 UGT members inten evolutionary groups that are likely related to glycosylation modifications with secondary metabolites in alfalfa. These findings help uncover pivotal regulatory mechanisms associated with secondary metabolism in plant yield and quality and contribute to genetic modification and breeding in alfalfa and other plant species.

## Introduction

Glycosyltransferases (GTs) are enzymes that utilize activated glycosyl donors as substrates to regulate biochemical properties and subcellular localization by catalyzing the glycosylation reactions of molecules such as proteins, lipids, plant hormones, and phenylpropane compounds (Song et al., 2018; Speeckaert et al., 2020). In plants, glycosylation can preserve the diversity of small molecular compounds and plays an important role in regulating biological processes such as seed germination, growth, flowering, fruiting, and senescence (Li H. et al., 2020; Wu et al., 2020). The GT family 1 (GT1), which is referred to as UDP glycosyltransferase (UGT), mainly catalyzes UDP sugar transfer to specific receptors, such as proteins, nucleic acids, antibiotics, alkaloids, and plant hormones, and is the largest glycosyltransferase gene family in the plant kingdom (Zhang et al., 2020).

UGT is ubiquitous existed in both unicellular algae, where it has a simple structure, and higher plants, where it has a complicated structure (Rehman et al., 2018; Song et al., 2018). The first plant UGT family gene, *Bronze1*, which encodes a UGT enzyme that can synthesize flavonoid glycosides and regulate the accumulation of melanin in maize (*Zea mays*) seeds, was identified by Barbara McClintock in 1977 when she was studying the genetic traits of transposons in maize (Dooner and Nelson, 1977). Currently, many UGT gene families have been identified in several crops and plant species at the whole genome-wide level, such as wheat (*Triticum aestivum*) (He et al., 2018), soybean (*Glycine max)* (Yano et al., 2018), rice (*Oryza sativa*) (Dong et al., 2020), and *Arabidopsis thaliana* (Chen T. T. et al., 2020). Meanwhile, the sophisticated roles of plant hormone regulation, biotic and abiotic stress adaptation, yield and quality enhancement, and secondary metabolism adjustment, that UGTs played, have also been highlighted (Mohnike et al., 2021; Kurze et al., 2022; Wang et al., 2022; Zhang et al., 2022).

For instance, UGTs such as UGT84B1 and UGT76F1 in *Arabidopsis* (Aoi et al., 2020; Chen L. et al., 2020), CsUGT85A53 in *Camellia sinensis* (Jing et al., 2020), UGT76C2 in *Arabidopsis* (Li Y. et al., 2020), and the promoter of *SbUGT* in poplar (*Scutellaria barbata*) (Li et al., 2019), reportedly participate in regulating the glycosylation modifications of auxin (IAA), abscisic acid (ABA), and cytokinin (CK), respectively, controlling the growth and development of the roots, flowering, and root buds, respectively. Furthermore, UGTs such as AtUGT71C3 can compromise the excessive defense response of *Arabidopsis* by regulating the steady state of MeSA and SA (Chen et al., 2019), while the down-regulation of *CsUGT91Q2* decreased the scavenging capacity of reactive oxygen species and caused sensitivity to low-temperature stress (Zhao et al., 2020). Moreover, UGTs such as GhUGT103, GhUGT105 (Xiao et al., 2019), and OsUGT83A1 (Dong et al., 2020) were involved in regulating fiber development in upland cotton (*Gossypium hirsutum* L.) and grain size in rice, and have been considered important traits of grain yield.

In particular, UGTs such as UGT79B2 and UGT79B3 in *Arabidopsis* (Li et al., 2017), ZmUFGT2 (UGT78D2) in maize (Li et al., 2018), and UGT87A1 in *Carex rigescens* (Zhang K. et al., 2021), are involved in secondary metabolism *via* the glycosylation of flavonoids, which are a kind of natural polyphenolic secondary metabolite with antioxidant and free radical scavenging functions in different tissues (Rojas Rodas et al., 2014; Huo et al., 2021). Additionally, these UGTs can catalyze glycosylation reactions from anthocyanins, kaemferol, and quercetin to the corresponding glycosides *in vivo*. Decreasing the expression of *CsUGT91Q2* resulted in the decrease of neroli glycoside and caused sensitivity to low-temperature stress (Zhao et al., 2020), indicating that UGT acts on glycosylation terpenoids, which are the largest category of secondary metabolites (with more than 40,000 structures) and have several biological functions such as anti-tumor, antibacterial, hypoglycemic and economic value due to their flavor, tastants, and colorants (Chan et al., 2016; Kurze et al., 2022). Moreover, studies have demonstrated that the soyasaponins of soybean (Yano et al., 2018) and rebaudioside A (Reb A) of *Stevia rebaudiana* (Kim et al., 2019) can be converted by UGT91H9 and UGT76G1 respectively, leading to better taste and higher quality. Generally, the glycosylation modification functions that plant UGTs have on various secondary metabolites can generate active molecules that are involved in several processes related to biological regulation (Rehman et al., 2018; Yu et al., 2022). Therefore, studying the functions of UGTs involved in secondary metabolism is essential for identifying and modifying genetic traits highly relevant to crop yield and quality production.

There is a highly conserved plant secondary product glycosyltransferase box (PSPG) composed of 44 amino acids at the C-terminal of the UGT gene family protein (Cheng et al., 2019). A previous study proposed that the whole folding and core region of UGT proteins in plants are conserved since the C-terminal domain of UGT proteins mostly recognizes the same or similar glycosyl donors, and the N-terminal domain recognizes specific receptors (Nair et al., 2020). This indicates that the PSPG motif is an important binding region that can recognize the UDP sugar donor of uridine, which contributes to glycosylating specific secondary metabolites in plants (Cheng et al., 2019). While the evolutionary relationships of the UGT gene family members are related and have similar exon and intron numbers and sequence characteristics (Xiao et al., 2019), 122, 148, 184, and 128 *UGT* genes have been identified in *Arabidopsis*, maize, grape (*Vitis vinifera* L.) and soybean, respectively, using the PSPG motif (Rehman et al., 2018). This suggests that UGTs have certain specificity in different plant species. For example, after comparing the amino acid sequences encoded by five *UGT* genes related to terpenoid glycosylation in *Panax notoginseng*, the positions of 17 amino acids were found to be highly conservative, while the other positions vary (Wang et al., 2020). Therefore, it is necessary to identify and characterize the specific UGT gene family in certain plant species before investigating the UGTs involved in secondary metabolic regulation.

Alfalfa (*Medicago sativa* L.) is the most important and widespread legume plant worldwide and is prized for its high protein content, nutritional quality, yield, and environmental adaptability (Barros et al., 2019; Shen et al., 2020). Studying the molecular regulatory role of *UGT* genes in alfalfa is important for improving its yield and quality through genetic engineering. However, few studies have researched *UGT* genes in alfalfa, and the biological role of UGTs associated with secondary metabolism has not been understood until now. Recently, the genome sequence data of the autotetraploid plant alfalfa has been gradually completed (Chen H. et al., 2020; Shen et al., 2020; Long et al., 2022), providing valuable genomic information and making it possible to identify and research the function of the UGT gene family in alfalfa at the genome-wide level. In this study, we performed a systematic genome-wide analysis to identify UGT family genes in alfalfa and screened 90 of them for their association with secondary metabolism. These 90 *UGT* genes were then clustered into ten phylogenetic groups, and their evolutionary relationship, conserved motif, chromosomal distribution, gene structure, *cis*-acting elements, intra- and inter-species collinearity, and tissue expression patterns were analyzed. This study aimed to identify the UGT gene family in alfalfa involved in secondary metabolism at the genome-wide level and clarify their gene and protein molecular characteristics, evolutionary relationship, and major induced factors. The results provide comprehensive genetic information and outline the molecular features of *UGT* genes related to secondary metabolism in alfalfa.

## Materials and methods

### Plant materials and treatments

Alfalfa (*Medicago sativa* L. cv. Zhongmu-1) seeds preserved in our lab were used as the plant material. Alfalfa seeds were germinated on filter paper immersed with ultrapure water in a petri dish in a growth chamber (GXZ-500, Jiangnan, China) 16 h light (1,200–1,250 μmol m^–2^ s^–1^) at 25°C and 8 h darkness at 20°C. After 7 days of germination, the seedlings were transferred to a plastic cuboid container (25 cm × 20 cm × 7.5 cm) with 1/2 Hoagland nutrient solution under controlled conditions. For the transcriptional expression assay, the 4-week-old Hoagland solution was used to culture alfalfa seedlings under identical growth conditions. Samples were obtained from seven sections, including the new leaves (NL), the mature leaves (ML), the first real leaves (FL), and the stems of each 1/3 position from top to bottom: upper stems (US), middle stems (MS) and bottom stems (BS), and root, with three biological replicates for each (Ma et al., 2021).

### Identification and sequence analysis of *UGT* genes in alfalfa

The *M. sativa* “Zhongmu No. 1” genome information (Shen et al., 2020) was downloaded from the figshare website^1^. UGT protein sequences of *Arabidopsis* were retrieved from the P450 database^2^. Alfalfa *UGT* genes were confirmed using the local BLAST with a cutoff E-value of 1.0 × 10^–10^ and TBtools software (Huazhong Agricultural University, Wuhan, China) (Chen C. et al., 2020). The UGT protein sequences of alfalfa were aligned using the Hidden Markov Model (HMM) model on the Pfam online website^3^ (Finn et al., 2014). The UDPGT domain (PF00201) was used to identify UGT members in the Pfam database^4^ (Finn et al., 2014).

UGT proteins identified in alfalfa were annotated in the eggNOG-Mapper database^5^ functional annotation enrichment (Huerta-Cepas et al., 2019). UGT proteins involved in secondary metabolism were considered by the Pathway Name and Description in the KEGG Pathway Database^6^ (Antonov et al., 2010), including metabolic pathways (K01115), glucosinolate biosynthesis (K11280), biosynthesis of various plant secondary metabolites (K21371), monoterpenoid biosynthesis (K21373, K21374), anthocyanin biosynthesis (K12930, K12938, K17193), zeatin biosynthesis (K13493, K13494, K13495, K13496), and phenylpropanoid biosynthesis (K12356).

### Phylogenetic analysis and comparison of *MsUGT* genes

Basic physical and chemical characteristics, including the sequence start position, sequence end position, length, molecular weight, isoelectric point, and Grand average of hydropathicity (GRAVY) of *UGT* genes in alfalfa, which were screened by KEGG functional enrichment, were obtained from the ExPASy website^7^ (Gasteiger et al., 2003). Subcellular localization prediction was performed by WoLF PSORT^8^ (Horton et al., 2007).

Divergent UGT protein sequences were removed from the identified sequences after alignment with MEGA 11 software. The phylogenetic tree was built using MEGA11 software (Tamura et al., 2021) with UGT protein sequences from alfalfa, *Arabidopsis*, and maize by the neighbor-joining (NJ) method (bootstrap values for 1,000 replicates) and was visualized using the Evolview website^9^. UGT proteins in alfalfa were classified according to the 14 reported evolutionary groups in *Arabidopsis* (A-N) and the other three new groups found in maize (O-Q). The conserved PSPG motif, composed of 44 amino acids in each UGT phylogenetic group, was analyzed using MEME software^10^ (Bailey et al., 2009).

### Chromosomal location and structural characterization analysis of *MsUGT* genes

Information on the chromosomal location of *UGT* genes was retrieved and generated by TBtools. Ten motifs with lengths of 15–50 amino acids in UGT proteins from alfalfa were identified using the online MEME Suite (see text footnote 10) (Bailey et al., 2009). The MAST file was obtained from the MEME website, and the Newick tree string was visualized in TBtools. The exon and intron structures of *MsUGT* genes were analyzed using TBtools software, inputting gene annotation GFF files and Newick tree string.

### Analysis of *cis-*acting elements of *MsUGT* genes

The promoter regions of *MsUGT* genes (2,000 bp sequence upstream of the DNA genome sequences) were obtained from the *M. sativa* genome database. PlantCARE database^11^ was used to predict the *cis*-acting elements (Lescot et al., 2002). The results containing promoter information and the phylogenetic evolution of the UGT gene family in alfalfa were visualized using TBtools software.

### Gene duplication pattern and collinearity analysis of *MsUGT* genes

The identification of gene duplication patterns in *MsUGT* genes was performed using multiple collinear scanning toolkits (MCScanX) with an E-value set to 10^–5^. The syntenic relationship between *MsUGT* genes and *UGT* genes from *Medicago truncatula*, *Arabidopsis thaliana*, *Oryza sativa*, and *Glycine max* were determined using the Dual Synteny Plotter function in the TBtools software. The necessary genome sequence was obtained from the BioMart Ensemble plants database^12^.

### Tissue differential expression analysis of *MsUGT* genes in each evolutionary group

Total plant RNA was extracted using the total RNA Extraction kit (Shanghai Promega Biological Products, China), according to the manufacturer’s instructions. Complementary DNA was generated with reverse transcriptase (TaKaRa, Beijing, China) at 42°C for 2 min, then at 37°C for 15 min, and finally at 85°C for 5 s. Quantitative real-time PCR (qRT-PCR) was conducted with 2 × EasyTaq^®^ PCR SuperMix (+ dye) (TransGen Biotech, Beijing, China), according to the manufacturer’s instructions, on the CFX96 Touch™ RT-PCR system (BioRad, Los Angeles, CA, United States). Three technical repeats were performed for each sample. The specific primer sequences of *MsUGT* genes for qRT-PCR determination are provided in Supplementary Table 1. The *Ms-ACTIN* gene was used as an internal control (Supplementary Table 1). The relative transcriptional expressions of each gene were analyzed based on the 2^–ΔΔ*Ct*^ method (Livak and Schmittgen, 2001). SPSS 20 software was used to process the experimental data by the analysis of variance (ANOVA). The least significant difference method (LSD, *P* < 0.05 level) was used for statistical testing (Ma et al., 2016). The results were completed and presented using Graphpad Prism 8.

## Results

### Identification and secondary metabolism pathway enrichments of *UGT* genes in alfalfa

**FIGURE 1:**
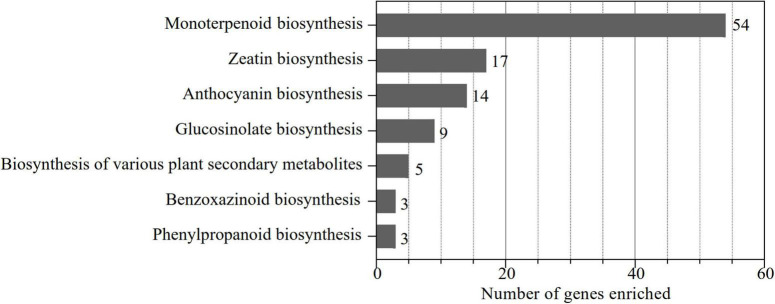
Number of *UGT* genes involved in secondary metabolism-related pathways. The KO identifiers (called K numbers) we considered associated with the secondary metabolism network are displayed: monoterpenoid biosynthesis (K21373, K21374), zeatin biosynthesis (K13493, K13494, K13495, K13496), anthocyanin biosynthesis (K12930, K12938, K17193), glucosinolate biosynthesis (K11280), biosynthesis of various plant secondary metabolites (K21371), phenylpropanoid biosynthesis (K12356), and benzoxazinoid biosynthesis (K13227).

To identify the UGT gene family in alfalfa, a local blast analysis was conducted on “Zhongmu No. 1” protein sequences using 122 UGT protein sequences from *Arabidopsis*. In total, 239 putative UGTs containing the UDPGT domain (PF00201) in alfalfa were obtained and were named according to their chromosomal location. In addition, these 239 *UGT* genes were enriched to KEGG pathways corresponding to the eggNOG-Mapper database (Figure 1). The results demonstrated that 90 genes were enriched in the functional pathways of monoterpenoid biosynthesis (54), zeatin biosynthesis (17), anthocyanin biosynthesis (14), glucosinolate biosynthesis (9), biosynthesis of various plant secondary metabolites (5), benzoxazinoid biosynthesis (3), and phenylpropanoid biosynthesis (3) (Figure 1), which are considered the pathways associated with secondary metabolism. This indicates that monoterpenoid biosynthesis is the dominant pathway associated with *UGT* genes in alfalfa, while zeatin biosynthesis, anthocyanin biosynthesis, and glucosinolate biosynthesis are the main functional pathways of enriched *UGT* genes. Therefore, based on these screening results, we analyzed the molecular characteristics, phylogenetic relationships, chromosomal localization, structural characterization, conserved motifs, *cis*-acting elements, intra- and interspecies collinearity, and expression pattern of these 90 UGT members to represent *UGT* genes related to secondary metabolism in alfalfa.

### Molecular characteristics of UGT members associated with secondary metabolism in alfalfa

To characterize the 90 UGTs in alfalfa screened above, the basic molecular characteristics of the proteins were analyzed. The gene name, gene ID based on the “Zhongmu No. 1” database, the start and end point, the number of amino acids, molecular weight (Mw), isoelectric point (pI), and GRAVY of each gene are displayed in Supplementary Table 2. The amino acid length of MsUGTs varied among the 90 proteins, from 82 to 1,683 amino acids. The theoretical pI and Mw ranged from 4.94 to 8.86 and from 9,097.77 Da to 188,543.37 Da, respectively. Analysis of predicting subcellular localization found that 47 MsUGT members were localized in the chloroplast; 27 and 9 MsUGTs were distributed in the cytoplasm and nucleus, respectively; while a small number of MsUGT proteins were widely located in the mitochondria (2), extracell (1), peroxisome (1) and plasma membrane (1). These results provide an integrative view of the molecular characteristics of these 90 UGT members.

### Phylogenetic analysis of UGT members associated with secondary metabolism in alfalfa

**FIGURE 2:**
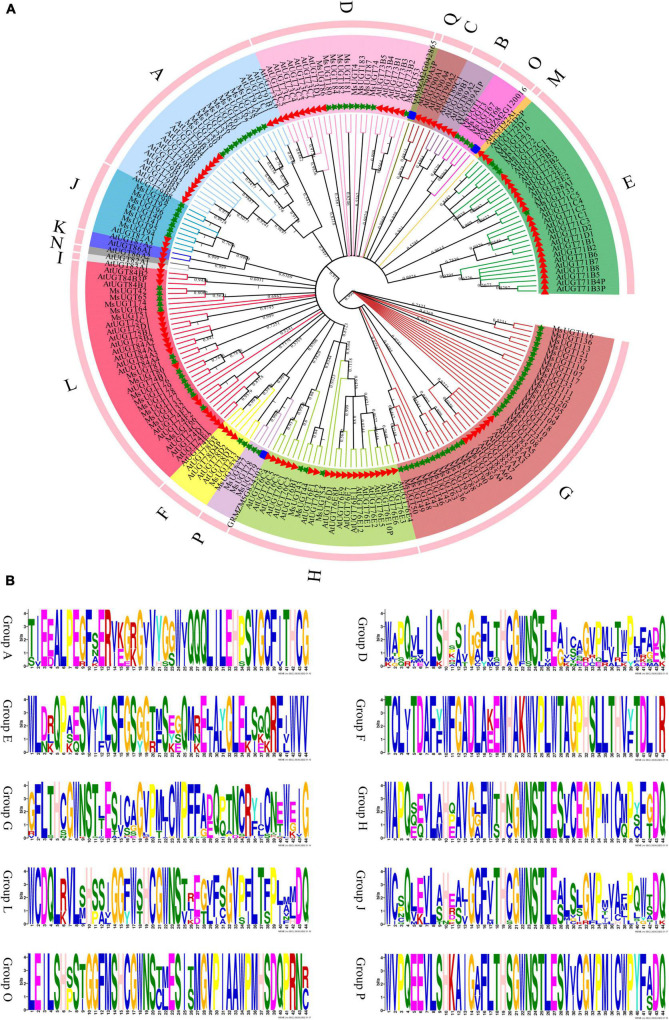
Phylogenetic and PSPG box analyses of MsUGT proteins associated with secondary metabolism in alfalfa. **(A)** The phylogenetic tree is generated by the NJ method with 1,000 repeats from the sequences of *Arabidopsis thaliana*, *Zea mays*, and *M. sativa*. **(B)** The PSPG motif of each phylogenetic group is shown.

To better understand the evolutionary relationship of the 90 *UGT* genes associated with secondary metabolism, phylogenetic analysis was conducted based on the amino acid sequence similarity and topological structure (Figure 2). The results demonstrated that the 90 UGT proteins were classified into ten different evolutionary groups (Figure 2A). The four groups with the largest numbers were G, L, A, and D, with 33, 14, 10, and 10 UGT members, respectively; the three groups with the fewest UGT proteins were groups F, H, and P, with 2, 3 and 3, respectively; and none were found in groups B, C, I, K, M, N, and Q in alfalfa. Moreover, ten phylogenetic groups of the *MsUGT* genes were further distinguished by the symbolic PSPG motif (Figure 2B). While the PSPG boxes were presented in each phylogenetic group, the PSPG sequences in different groups were not completely identical (Figure 2B). Twenty-two and 32 amino acid residues of PSPG motifs were fully conserved in groups D, J, and L and in groups H and P, respectively. Additionally, 16 amino acid residues were the same in these sequences, and groups A, E, F, G, and O had low sequence similarity with other groups, though they were conservative within the group.

### Chromosomal location and structural characterization of UGTs associated with secondary metabolism in alfalfa

**FIGURE 3:**
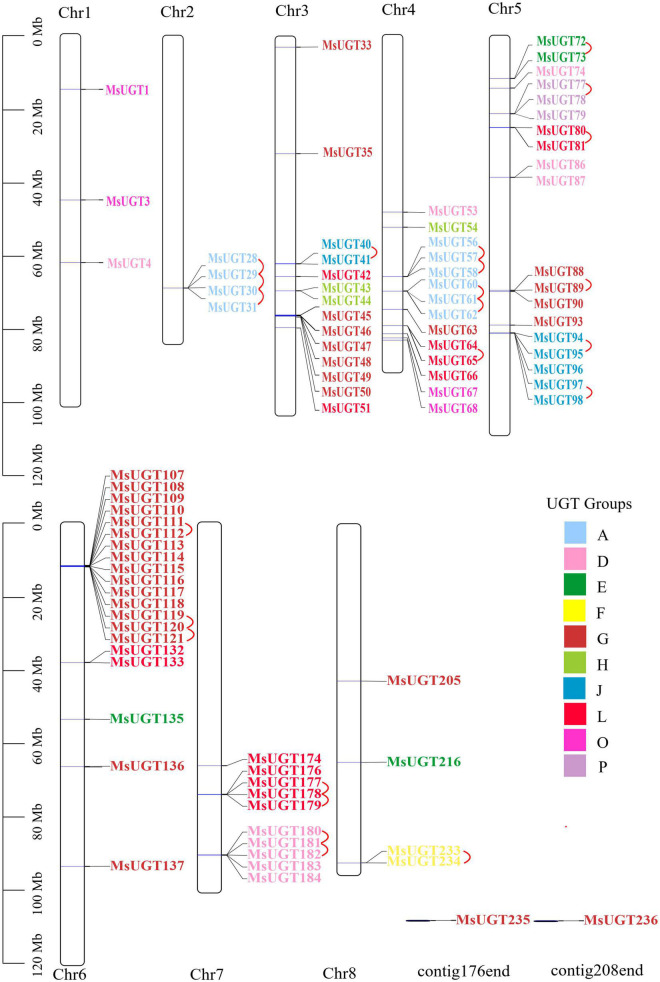
Chromosomal location and tandem duplication event analyses of *MsUGT* genes associated with secondary metabolism in alfalfa. The tandem duplicated gene pairs are connected by red lines. Each phylogenetic group is indicated by the same color used in the evolutionary tree in Figure 2A.

To explore the genomic distribution of alfalfa *UGT* genes, the chromosomal location of *MsUGT* genes was investigated based on the genome annotation database (Figure 3 and Supplementary Table 3). As shown in Figure 3, 88 UGTs were unevenly distributed across eight chromosomes, and two UGTs were identified in the scaffolds. Twenty *UGTs* were distributed on chromosome 6, and 19, 14, and 14 *UGTs* were distributed on chromosomes 5, 4, and 3. Notably, 23 gene pairs were involved in tandem duplications covering 39 *UGT* genes, which were clustered into seven alfalfa chromosomes (Figure 3).

**FIGURE 4:**
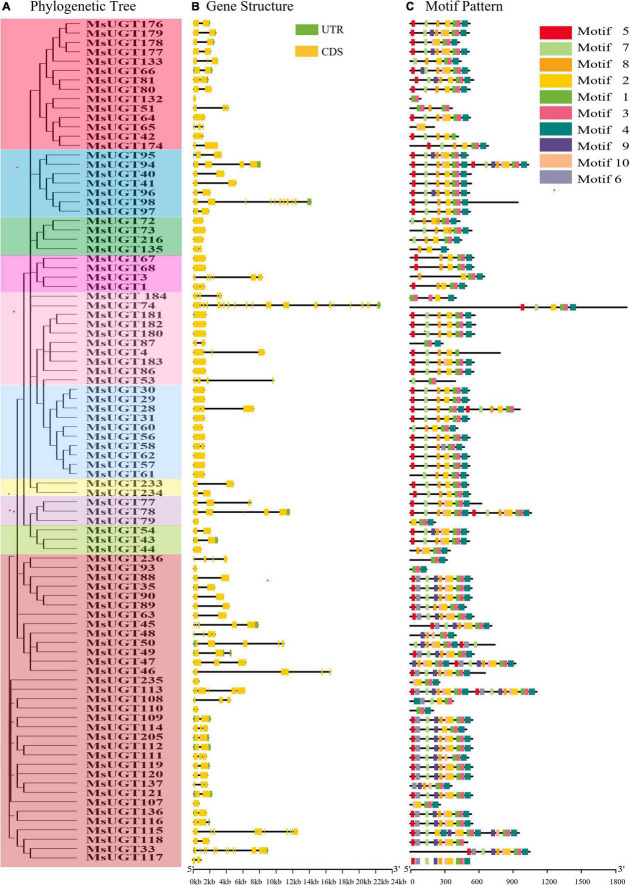
The phylogenetic relationship, exon-intron structure, and conservative motif analysis of 90 *UGT* genes associated with secondary metabolism in alfalfa. **(A)** The phylogenetic tree of 90 MsUGTs was constructed by the NJ method with 1,000 repeats. **(B)** The exon-intron structure analysis of 90 *UGT* genes was represented in detail. **(C)** The conservative motif analysis of 90 MsUGTs was predicted. Each phylogenetic group is indicated by the same color used in the evolutionary tree in Figure 2A.

Analysis of the gene structure and motifs was constructed with the phylogenetic tree (Figure 4A). Gene structure analysis demonstrated that the intron numbers of *UGT* genes associated with secondary metabolism ranged from 0 to 23 (Figure 4B). Most *UGT* genes contained 0–2 introns, *MsUGT74* had 23 (the most), and 26 genes (*MsUGT29*, *30*, *31*, *44*, *56*, *57*, *60*, *61*, *62*, *64*, *67*, *68*, *72*, *73*, *79*, *86*, *93*, *107*, *110*, *132*, *180*, *181*, *182*, *183*, *216*, and *235*) lacked introns (Table 1). We also identified ten motifs with 15–50 amino acids (Figure 4C and Supplementary Table 4). Most UGT proteins contained 6–10 conserved motifs, and UGT proteins of the same branch had similar conserved motif composition and sorting order, suggesting that MsUGT proteins in the same group could share similar functions.

**TABLE 1:** Number of *MsUGT* genes in each group according to number of introns.

Group	No. of introns										Total
	0	1	2	3	4	7	9	10	12	23	
A	8	1	1	-	-	-	-	-	-	-	10
D	5	1	1	-	2	-	-	-	-	1	10
E	3	1	-	-	-	-	-	-	-	-	4
F	-	2	-	-	-	-	-	-	-	-	2
G	4	16	5	3	3	-	1		1	-	33
H	1	2	-	-	-	-	-	-	-	-	3
J	-	5	-	1	-	-	-	1	-	-	7
L	2	8	4	-	-	-	-	-	-	-	14
O	2	1	-	-	-	1	-	-	-	-	4
P	1	-	-	1	1	-	-	-	-	-	3
Total	26	37	11	5	6	1	1	1	1	1	90

“-” represents absent.

### Analysis of *cis*-acting elements of *UGT* genes associated with secondary metabolism in alfalfa

**FIGURE 5:**
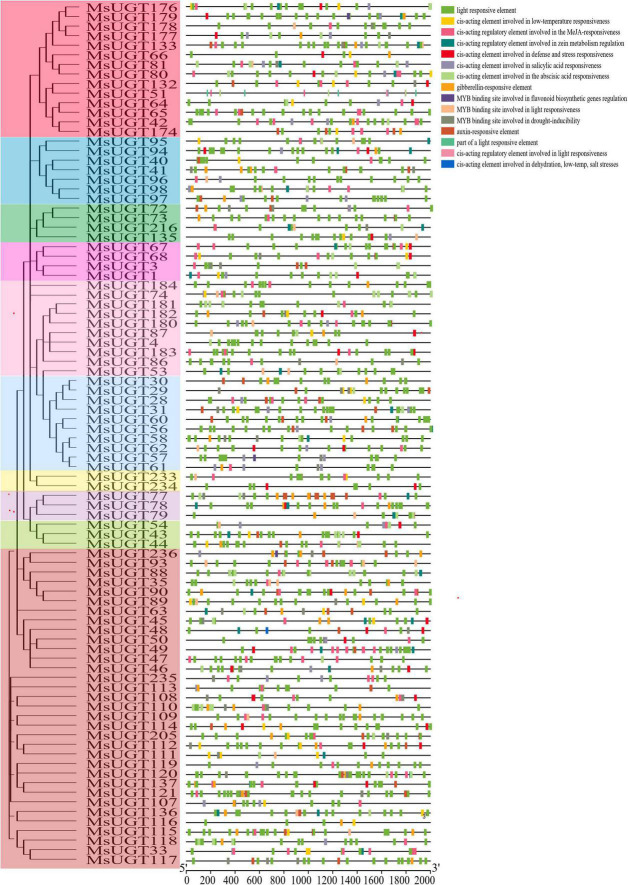
*Cis*-acting elements analysis in the promoter region of *UGT* genes associated with secondary metabolism in alfalfa. Each phylogenetic group is indicated by the same color used in the evolutionary tree in Figure 2A.

To better investigate the characteristics of *UGT* promoters, the *cis*-acting elements were analyzed (Figure 5 and Supplementary Table 5). The results demonstrated that the *cis*-acting elements of promoter regions of the UGT gene family were classified into five categories, including light responsiveness, plant hormone response, resistant response, promoter core elements (TATA-box), and common *cis*-acting element (CAAT-box). Except for the promoter core elements and common cis-acting elements, the number of light-responsive elements was the largest, with 2,676. Phytohormone-responsive elements were primarily related to GA, MeJA, IAA, ABA, and SA, and data for three abiotic stress response elements such as drought, low temperature, and salt stress, are displayed in detail (Figure 5). *MsUGTs* contained 11 flavonoid biosynthesis-related elements due to the potential involvement of *MsUGT* genes in secondary metabolism. Overall, the *cis*-element analysis indicated that *UGT* genes could respond to different kinds of metabolic processes.

### Gene duplication and collinearity analysis of *UGT* genes associated with secondary metabolism in alfalfa

**FIGURE 6:**
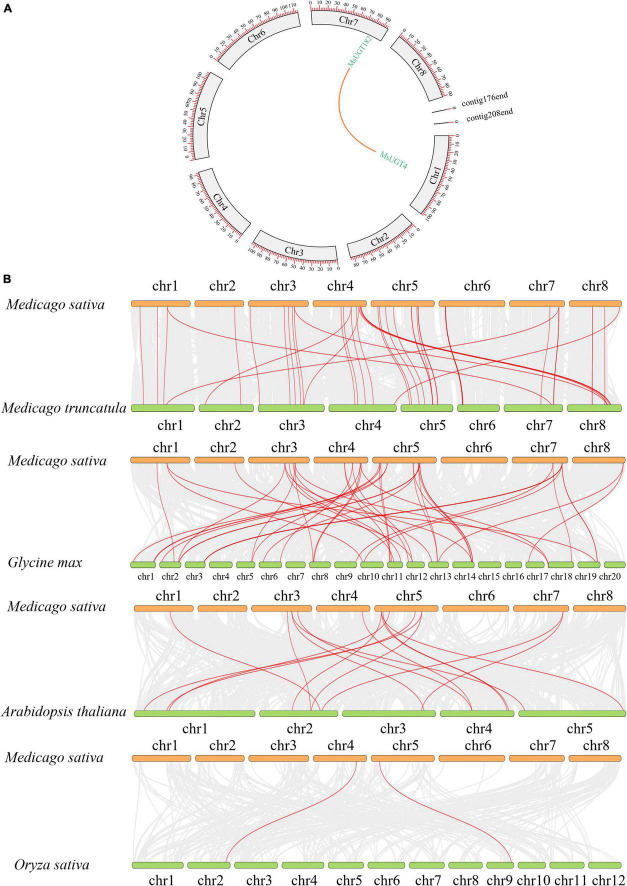
*MsUGT* gene duplications in the genomes between and within species. **(A)** The duplicated event is a collinearity block in the alfalfa genome, and the segmental duplication between *MsUGT4* and *MsUGT182* is drawn with an orange line. **(B)** The collinear blocks of *UGT* genes between *M. sativa* and *M. truncatula*, *Glycine max*, *Arabidopsis thaliana*, and *Oryza sativa*, respectively, are indicated by red lines.

To explore the collinearity of the MsUGT gene family between and across species, we constructed syntenic maps of *MsUGT* genes with those in *M. truncatula* and three other model plants of *Arabidopsis thaliana*, *Glycine max*, and *Oryza sativa* (Figure 6 and Supplementary Table 6). A segmental duplication event was identified from *MsUGTs* (*MsUGT4*/*MsUGT182*), which was distributed on chromosomes 1 and 7 (Figure 6A). A total of 118 corresponding orthologs were identified, including 55, 16, 45, and 2 genes from *M. truncatula*, *Arabidopsis*, soybean, and rice, respectively (Figure 6B). These genes were unevenly distributed on eight chromosomes in alfalfa, while chr3 and chr5 identified more collinear genes than the others. These results indicate that *MsUGT* genes displayed different numbers of syntenic lines with four species, while most paralogous *MsUGTs* pairs could have variable expression patterns.

### Tissue differential expression analysis of *UGT* genes associated with secondary metabolism in alfalfa

**FIGURE 7:**
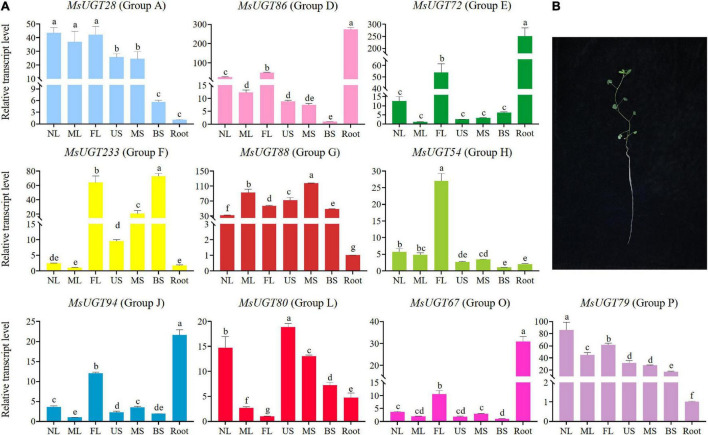
Transcriptional expression analysis of Ms*UGT* genes of 10 groups in seven different tissues. **(A)** Genes of *MsUGT28*, *86*, *72*, *233*, *88*, *54*, *94*, *80*, *67*, and *79* were selected from groups A, D, E, F, G, H, J, L, O, and P, respectively. The gene expression represented in each evolutionary group is shown with the same color as the phylogenetic tree in Figure 2A. **(B)** Tissues are harvested for transcriptional expression. NL, new leaves; ML, mature leaves; FL, the first real leaves; US, Upper stems; MS, middle stems; BS, bottom stems; Root.

To investigate the tissue expression profiles of the 90 *UGT* gene members associated with secondary metabolism in alfalfa, we performed a transcriptional expression analysis of one representative gene from each evolutionary group (Figure 7 and Supplementary Table 7). The results demonstrated that the expression of *MsUGT28*, *MsUGT54*, and *MsUGT79* from groups A, H, and P have similar tissue expression profiles and were highest in the leaves and lowest in the roots (Figure 7A and Supplementary Table 7). *MsUGT28* expression in leaves was significantly higher than in the roots and stems, and the highest expression occurred in NL, reaching 43.52; the expression of *MsUGT79* was similar to that of *MsUGT28*, while its highest expression level appeared in NL, reaching 86.02, and its expression in three stem tissues also reached 17.21, 27.74 and 31.71; the expression level of *MsUGT54* reached 27.03, 5.64, and 4.80 in FL, NL, and ML, respectively, which was significantly higher than in the stems and roots.

The expression of *MsUGT86*, *MsUGT72*, *MsUGT94*, and *MsUGT67* from groups D, E, J, and O in the roots reached 273.23, 250.60, 21.58, and 30.89, respectively, which was higher than in other tissues. Of these, the expression of *MsUGT86* in three stem tissues from the bottom to the top was the lowest, and the expression in the leaves exceeded that in the stems, reaching 24.59, 12.27, and 48.53, respectively. The tissue expression patterns of *MsUGT72*, *MsUGT94*, and *MsUGT67* in FL were 53.79, 12.02, and 10.52, which was significantly higher than in other leaves and stems.

However, some *UGT* genes in each evolutionary group showed specific tissue expression profiles, such as *UGT88*, *UGT233*, and *UGT80* from groups G, F, and L, respectively. The expression of *MsUGT88* in group G exceeded 30 in both the stems and leaves, which was much higher than in the roots. The expression of *MsUGT233* in group F showed the highest expression in FL and BS, with 63.86 and 72.9, respectively, while MS was 20.48, which was significantly higher than in NL, ML, US, and root tissues. The expression of *MsUGT80* in group L was the highest in US, 18.86, and decreased from NL and MS, BS, root, ML, and FL. In summary, *MsUGT28* in group A, *MsUGT54* in group H, and *MsUGT79* in group P had the highest expression in leaves; *MsUGT233* in group F, *MsUGT88* in group E, and *MsUGT80* in group L had the highest expression in stem tissues; *MsUGT86* in group D, *MsUGT72* in group E, *MsUGT94* in group J, and *MsUGT67* in group O had the highest expression in the roots. These results demonstrate that *MsUGT* genes related to secondary metabolism could function in the full plant, and that *MsUGT* genes in each evolutionary group had tissue-specific expression profiles.

## Discussion

Alfalfa is the most important legume in the world due to its high yield, high quality, and environmental adaptability, while the glycosylation reactions catalyzed by UGTs are necessary for maintaining and enhancing metabolic homeostasis by regulating flavonoids, terpenoids, lignin, and other metabolites. Accordingly, identifying and characterizing the *UGT* genes involved in secondary metabolism is important for improving yield, quality, and stress tolerance traits in alfalfa. In this study, 90 *UGT* genes associated with secondary metabolism screened by KEGG pathway functional enrichment were identified in alfalfa. They were classified into ten evolutionary groups, and their basic molecular characteristics, evolutionary genetic information, gene structure, motif conservation, promoter region function prediction, intra- and inter-species collinearity, and expression patterns were analyzed. Based on these results, their basic molecular features, genetic evolution properties related to secondary metabolism, and *cis*-element analysis were assessed.

In our study, *MsUGT* genes associated with secondary metabolism had similar conservative motifs and gene structures, demonstrating that the number of genes containing 0–2 introns adds up to 74 and accounts for 82.2% to the 90 members. The gene structures were similar to the results of previous studies of *UGT* genes in upland cotton (Xiao et al., 2019), *Arabidopsis* (Wu et al., 2020), and pomelo (*Citrus grandis*) (Wu et al., 2020). Of the 36 UGT members of upland cotton (Xiao et al., 2019), 17 and 19 genes contained one and two introns, respectively, while approximately 50% of the genes encoding glycosyltransferase in *Arabidopsis* and pomelo had no introns, most remaining *UGTs* contained one or two introns, and few genes contained multiple introns (Wu et al., 2020). The motif sequences of UGT members in groups with evolutionary relationships were similar, indicating that similarities in the motif location and gene structure of *MsUGTs* in the same subfamily sustained the phylogenetic classification of MsUGTs.

Our data also demonstrated that of the identified 90 *UGT* genes, 23 tandem duplications primarily occurred on chromosomes 2, 4, 5, 6, and 7, and one segmental duplication occurred on chromosomes 1 and 7. It is speculated that the variation in the number of UGT family members can be influenced by gene duplication events (Zhang H. et al., 2021). The tandem and segmental duplications of the genome provided more gene copies for generating novel gene functions and expanding gene families in alfalfa. However, compared with the seven segmental duplications in sacred lotus (*Nelumbo nucifera*) (Li H. et al., 2020), 24 gene segmental duplication events in cassava (*Manihot esculenta* Crantz) (Wu et al., 2021), and 28 segmental duplications in *Broussonetia papyrifera* (Wang et al., 2021), alfalfa has fewer segmental duplication genes and only one pair of highly homologous genes involving *MsUGT4* and *MsUGT182*. This indicates that tandem duplication events are more crucial than segmental duplications for *MsUGT* expansion. Furthermore, compared with the 16 and 2 corresponding orthologs identified in *Arabidopsis* and rice, the number of corresponding orthologs in *M. truncatula* and soybean was higher (36 and 40), demonstrating that UGT families could be related to the evolution of dicots but not monocots, and that whole genome duplication events could have contributed more to the evolution of species within the same family (Zhang H. et al., 2021).

The 90 *UGT* genes were clustered into ten evolutionary groups after confirming the UGT gene families in *Arabidopsis* and maize. Of them, ten genes (*MsUGT4*, *53*, *74*, *86*, *87*, *180*, *181*, *182*, *183*, and *184*) were identified in group D in alfalfa, which has been confirmed to catalyze terpenes and flavonoids; four UGTs (*MsUGT72*, *73*, *135*, and *216*) were identified in group E, which catalyzes phenylpropanoids (Song et al., 2019). For example, the UGT 72 family in group E has been shown to glycosylate two classes of phenylpropanoids (monolignols and flavonoids) (Speeckaert et al., 2022). Moreover, PSPG has been highlighted to play a critical role in regulating secondary plant metabolites (Zhang et al., 2018; Wang et al., 2020), and the PSPG analysis performed on the 90 MsUGT proteins in all ten evolutionary groups demonstrated that each evolutionary group had PSPG box in alfalfa. Accordingly, positions 1 (W), 4 (Q), 10 (H), 14 (G), 16 (F), 19–24 (HCGWNS), 27 (E), 32 (G), 34 (P), 39 (P), 43 (E/D) and 44 (Q) were highly conserved in MsUGT members in group D, J, and L, which indicate that crucial amino acids act on triterpenoids in *Panax notoginseng* (Wang et al., 2020). Therefore, differences in the glycosylation reactions of secondary metabolites among the evolutionary groups and the specificities in PSPG motif analyses further verified the involvement of these 90 *UGT* genes in secondary metabolic biosynthesis.

Our analysis demonstrated that ten *UGT* genes from each group showed different expression patterns in alfalfa tissues. Some MsUGT members were preferentially expressed in tissues as the stem, root, or leaves, which could be related to the higher accumulation of flavonoids and terpenoids. For instance, *MsUGTs* in groups A, H, and P, and *UGT* genes in groups D, E, J, and O have higher expression in leaves and root tissues, respectively, which is where flavone glucuronides primarily accumulate (Adiji et al., 2021). The highest expression levels of *MsUGT233*, *MsUGT88*, and *MsUGT80* were in BS, MS, and US, respectively, which are responsible for the glycosylation of the secondary metabolite lignin (Lin et al., 2016). This indicates that the expression of *UGTs* associated with secondary metabolism could differ widely from the specific active sites of glycosylation actions.

A remarkable amount of phytohormone response elements were obtained in the promoter regions of the 90 Ms*UGT* genes, suggesting that glycosylation reactions could be induced by these phytohormones. Plant hormones play an important role in responding to various stresses and normal growth and developmental progress (Tian et al., 2017; Radojicic et al., 2018; Shu et al., 2018). UGTs, such as UGT84A2 (Zhang et al., 2017), UGT75B1 (Chen T. T. et al., 2020) and UGT76D1 (Huang et al., 2018) in *Arabidopsis* could participate in the glycosylation of IAA, ABA, and SA, respectively, and regulate flowering, stress resistance, and systemic acquired immunity. However, recent studies have proposed that plant hormones could complete their functional role connected with some secondary metabolites by the UGTs. For example, GSA1 in rice had glycosyltransferase activity on flavonoids and lignitol monomers, indirectly affecting flavonoid-mediated auxin polar transport and the expression of related genes (Dong et al., 2020). Therefore, this might be an important line of UGT research in future studies.

In this study, eleven flavonoid response elements were identified in the promoter element, indicating that some UGTs in alfalfa could also be induced by flavonoids. Flavonoids are multifunctional secondary metabolites that are important for plants and human health, which can effectively delay antioxidation and anti-inflammatory effects in the human body (Yin et al., 2017a). A previous study demonstrated that CsUGT75L12 in tea plants can transfer sugar molecules to the C7 hydroxyl group of flavonoids, catalyzing the generation of flavonoid 7-*O*-glycosides (Dai et al., 2017); Plant secondary metabolites can be coupled with hormones to play an important role in responding to growth and developmental processes (Dong et al., 2020). The final step in the biosynthesis of particular flavonoids was glycosylation modifications by UGTs, while changes in the bioactivity, stability, and solubility of flavonoid compounds could ultimately affect the quality, yield, and adaptation of legumes to environmental changes (Yin et al., 2017a,b).

## Conclusion

In this study, we identified 239 *MsUGT* genes from the alfalfa genome and further screened 90 of them that are involved in secondary metabolic pathways. The basic molecular characteristics, motifs, gene structure, and duplication events of the 90 *MsUGT* genes were assayed and demonstrated. The genetic evolution properties, including group classifications and PSPG motifs related to secondary metabolism, were analyzed. Tissue differential expression analysis revealed the tissue-specific expression patterns of *MsUGT* genes in each evolutionary group. *Cis*-elements analysis of the promoter regions predicted that UGTs could be induced by various phytohormones and flavonoids. Therefore, our study provides comprehensive genetic information and molecular features of the *UGT* genes associated with secondary metabolism in alfalfa, which helps reveal some of the regulatory mechanisms associated with secondary metabolites that can improve yield and quality in alfalfa and other plant species.

## Data availability statement

The datasets presented in this study can be found in online repositories. The names of the repository/repositories and accession number(s) can be found in the article/Supplementary material.

## Author contributions

AY and XZ collected plant materials. AY and XJ performed the experiment. AY, XJ, LL, and LH analyzed the data. AY, XJ, and QH processed the figures and tables. YS, RL, and ML conceived and designed the experiment. AY, RL, and ML wrote the manuscript. AY, JK, LC, QY, RL, and ML revised and finalized the manuscript. All authors have read and approved the final manuscript.

## Footnotes

1
https://figshare.com/articles/dataset/Medicago_sativa_genome_and_annotation_files/12623960

2
http://www.p450.kvl.dk/UGT.shtml

3
http://hmmer.org/

4
https://www.ebi.ac.uk/Tools/pfa/pfamscan/

5
http://eggnog-mapper.embl.de/

6
https://www.genome.jp/kegg/pathway.html

7
https://web.expasy.org/compute_pi/

8
https://wolfpsort.hgc.jp/

9
http://www.evolgenius.info/evolview/

10
https://meme-suite.org/meme/tools/meme

11
https://bioinformatics.psb.ugent.be/webtools/plantcare/html/

12
https://plants.ensembl.org/index.html
